# The Clinical Frailty Scale as predictor of overall survival after resection of high-grade glioma

**DOI:** 10.1007/s11060-022-04001-y

**Published:** 2022-04-25

**Authors:** Julia Klingenschmid, Aleksandrs Krigers, Daniel Pinggera, Johannes Kerschbaumer, Claudius Thomé, Christian F. Freyschlag

**Affiliations:** Department of Neurosurgery, Medical University of Innsbruck, Anichstrasse 35, 6020 Innsbruck, Austria

**Keywords:** Frailty, Clinical frailty scale, Glioblastoma, Neurooncological surgery

## Abstract

**Background:**

The Clinical Frailty Scale (CFS) describes the general level of fitness or frailty and is widely used in geriatric medicine, intensive care and orthopaedic surgery. This study was conducted to analyze, whether CFS could be used for patients with high-grade glioma.

**Methods:**

Patients harboring high-grade gliomas, undergoing first resection at our center between 2015 and 2020 were retrospectively evaluated. Patients’ performance was assessed using the Rockwood Clinical Frailty Scale and the Karnofsky Performance Scale (KPS) preoperatively and 3–6 months postoperatively.

**Results:**

289 patients were included. Pre- as well as postoperative median frailty was 3 CFS points (IqR 2–4) corresponding to “managing well”. CFS strongly correlated with KPS preoperatively (r = − 0.85; p < 0.001) and at the 3–6 months follow-up (r = − 0.90; p < 0.001). The reduction of overall survival (OS) was 54% per point of CFS preoperatively (HR 1.54, CI 95% 1.38–1.70; p < 0.001) and 58% at the follow-up (HR 1.58, CI 95% 1.41–1.78; p < 0.001), comparable to KPS. Patients with IDH mutation showed significantly better preoperative and follow-up CFS and KPS (p < 0.05). Age and performance scores correlated only mildly with each other (r = 0.21…0.35; p < 0.01), but independently predicted OS (p < 0.001 each).

**Conclusion:**

CFS seems to be a reliable tool for functional assessment of patients suffering from high-grade glioma. CFS includes non-cancer related aspects and therefore is a contemporary approach for patient evaluation. Its projection of survival can be equally estimated before and after surgery. IDH-mutation caused longer survival and higher functionality.

## Introduction

Frailty is a term that is widely used to describe an individual’s vulnerability to develop increased dependency and mortality when exposed to a stressor. It represents a syndrome which is characterized by diminished strength, endurance and reduced physiologic function [1]. This can occur as the result of disease or diverse medical conditions.

The Clinical Frailty Scale (CFS) was developed by Rockwood et al. to objectively quantify the concept of frailty of an individual patient [2]. This tool is easy to use, as it is based on clinical judgement and therefore is recognized by physicians. This scale was initially introduced in geriatric medicine, where frailty manifests in sarcopenia, abnormal inflammatory and endocrine function as well as poor energy regulation [3, 4]. In intensive care medicine, frailty has been identified as a predictor for long-term mortality [5] and in orthopaedic surgery it serves to forecast unplanned repeat operations and consequent morbidity [6]. A CFS ≤ 4 is considered to describe patients that are ‘non-frail’. At the same time, evidence on frailty in neuro-oncological patients is limited. Whereas the Karnofsky Performance Scale (KPS) is routinely applied in oncology (and neurooncology) to assess patients’ functional impairment. The KPS was first published in 1949 and has been initially developed to evaluate a patient’s suitability for chemotherapeutic treatment against cancer [7]. It correlates with the patient’s individual tolerability and toxicity, as well as mortality during systemic therapy [8]. For decades, decision-making for neuro-oncological patients has been based on KPS [9–11].

Molecular characteristics of gliomas with isocitrate dehydrogenase (IDH) being one of the most prominent markers. According to the WHO classification of central nervous system tumors and the cIMPACT-NOW update 3, IDH wild-type astrocytomas now are considered to be corresponding to CNS WHO grade 4 tumors, if one of the following alterations is confirmed: high-level EGFR amplification, the combination of whole chromosome 7 gain and 10 loss or TERT promoter mutation [12, 13]. Thus, genetic features play the major role in diagnosis of brain tumors and will lead to an increase in CNS WHO grade 4 tumors provides robust data on the prognostic significance concerning overall (OS) and progression free survival (PFS) [14–16]. Further, it has been shown that the IDH mutational status can be strongly influencing the neuropsychological symptomatology of patients with glioblastoma [17, 18].

Neurooncological surgery faces challenges in treatment decisions due to an aging population and a reliable score for prognostication of postoperative functional status would be welcome. Therefore, the aim of this study was to investigate whether CFS can be applied in neuro-oncological patients and compare them to KPS, regarding estimation of their postoperative functional outcome.

## Materials and Methods

All patients with histopathologically confirmed high-grade glioma (HGG) including anaplastic glioma (WHO°III) and glioblastoma (GBM, WHOIV) who underwent their first surgical resection in our department between 2015 and 2020 were selected from the neuro-oncological database. In total, 310 patients were screened for eligibility, whereas 21 patients had to be excluded from final analysis: 9 due to missing preoperative KPS and 13 due to insufficient neuropathological data.

**Fig. 1:**
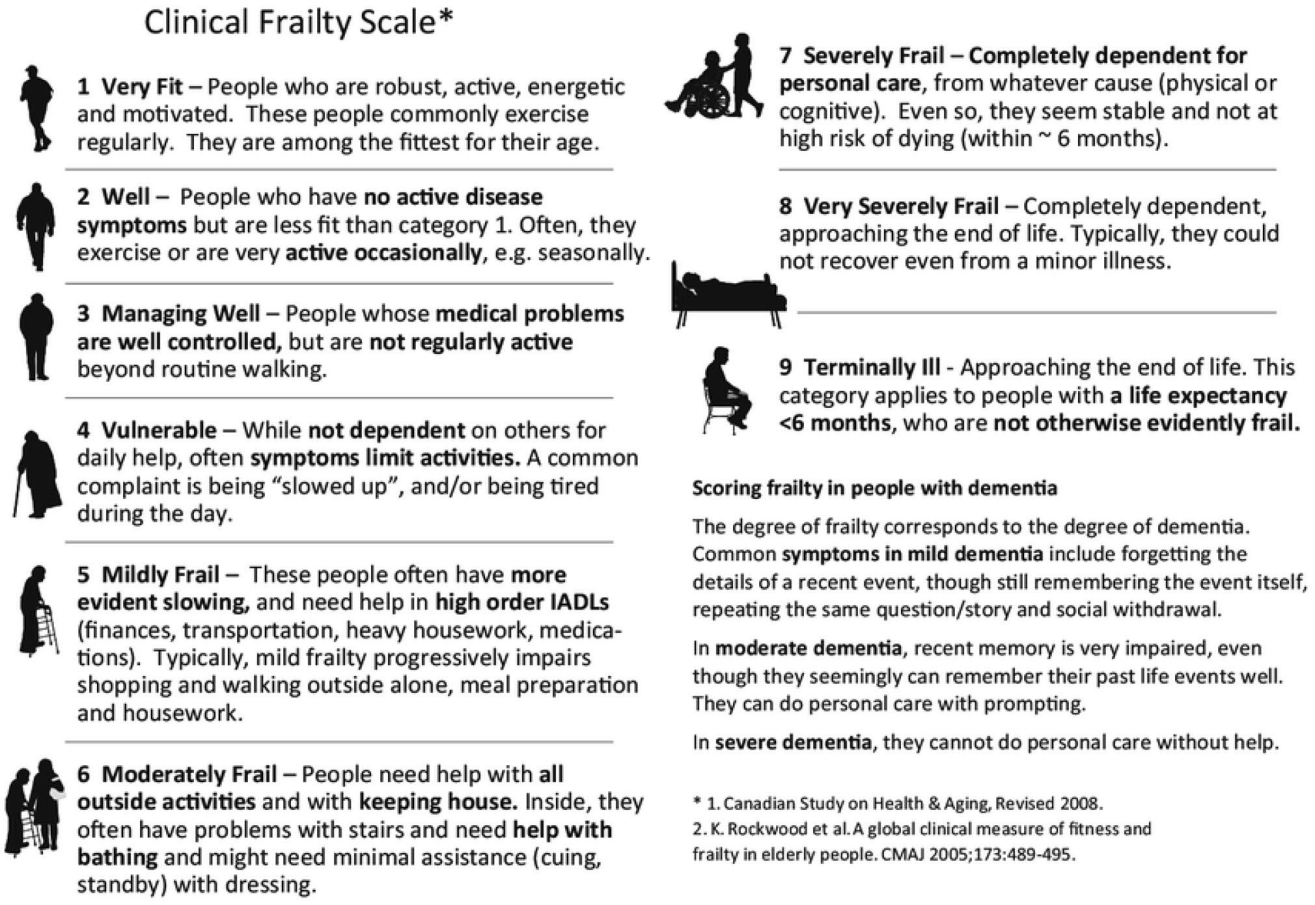
The Clinical Frailty Scale (CFS) by Rockwood et al. [2]

Frailty was rated before the resection and at the follow-up visit 3 to 6 months after the surgery using the Rockwood Clinical Frailty Scale (CFS), which consists of 9 levels: reaching from 1 being the most favourable (“very fit”) to 9 representing the least favourable score (“terminally ill”) (see Fig. 1) [2]. CFS was assessed retrospectively by four authors blinded to the outcome data using the functional description and standardized neurological status of the patients, which were documented in patients’ charts. Interrater variability was determined using the weighted kappa test..Karnofsky Performance Status Scale (KPS) was prospectively assessed in all patients preoperatively and 3 to 6 months after surgery as an institutional routine clinical standard. KPS consists of 10 levels divided into steps of 10 units reaching from 100 (no symptoms) to 0 (dead) [7].

Epidemiological data and routine neuropathological findings were also collected from our neuro-oncological database. Integrated neuropathological reports and WHO grading were based on the 4^th^ revised WHO classification system of CNS tumors [19, 20]. Immunohistochemistry (IHC) was applied to evaluate the R132H IDH1 mutation. If IDH remained wildtype, DNA sequencing was done for patients under 40 years for confirmation. Nuclear ATRX and EGFR expression were assessed with IHC. MGMT promotor methylation was evaluated using DNA sequencing with a cut-off at 8%. The time from imaging based diagnosis of suspected HGG to first resection was noted.

Statistical analysis and graphics were processed using IBM SPSS Statistics (IBM SPSS Statistics for Mac OS, Version 26.0. Armonk, NY: IBM Corp.). Kolmogorov–Smirnov test and histograms were used to check the normal distribution of scale data. If normal distribution was not confirmed, Mann–Whitney U-test for unpaired or Wilcoxon Signed Rank test for paired ranked or scale parameters was used. Correlations of non-parametric data were assessed by the Spearman’s test. In Cox regression analysis, hazard ratios for death considering independent parameters were revealed. Kaplan–Meier processing was applied to evaluate the overall survival. Analytical results with p < 0.05 were considered statistically significant.

The study was approved by the ethics committee of Medical University of Innsbruck (1333/2021). It was performed in accordance with the ethical standards as laid down in the 1964 Declaration of Helsinki and its later amendments or comparable ethical standards.

## Results

A total of 289 patients (171 male (59%) and 118 (41%) female) were included in this study. In 241 patients (83%) the HGG was diagnosed as WHO grade 4 and in 48 (17%) as grade 3. Age ranged from 8 to 88 years with a median of 62 years (IqR 51–73). Median time from the imaging-based diagnosis to surgery was 9 days (IqR 5–15). Kaplan–Meier processing showed a median overall survival of 14.5 months (IqR 12.4–16.6).

**Fig. 2:**
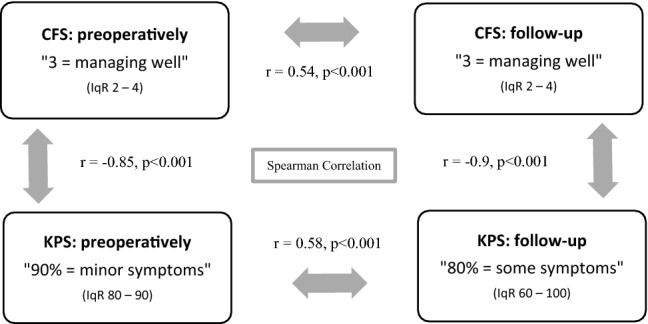
The Rockwood Clinical Frailty Scale (CFS) and Karnofsky Performance Score (KPS) before and 3–6 months after resection are shown as median with interquartile ranges. The Spearman correlation coefficient is noted between corresponding parameters. CFS and KPS correlated strongly with each other preoperatively and at the follow-up visit. There was only a moderate correlation between preoperative and follow-up CFS, as well as between preoperative and follow-up KPS, respectively. NB: Since CFS scores range from 1 to 9 (best to worst) and KPS scores range from 0 to 100 (worst to best), Spearman correlation (r) between two different scales is negative

Patients showed a median frailty score of 3 points (“managing well”, IqR 2–4) pre- as well as during the follow-up 3 to 6 months postoperatively. Median preoperative KPS was 90% (IqR 80–90), whereas median KPS in 3 to 6 months postoperative follow-up was 80% (IqR 60–100). The correlations between both clinical scales pre- and postoperatively are shown in Fig. 2. According to the Wilcoxon rank test, CFS and KPS were significantly worse at follow-up 3 to 6 months after surgery, compared to the presurgical scoring (p < 0.001 for each). Interrater variability was checked for and a high coherence of CFS was found to upon the four raters with kappa 0.81 (p < 0.001).

Patients’ age only mildly correlated with preoperative CFS (Spearman r = 0.27, p < 0.001) and KPS (Spearman r = − 0.30, p < 0.001) as well as postoperative follow-up CFS (Spearman r = 0.21, p < 0.01) and KPS (Spearman r = − 0.35, p < 0.001). The same applied for WHO grade, which mildly correlated with preoperative CFS (Spearman r = 0.29, p < 0.001) and KPS (Spearman r = − 0.33, p < 0.001) as well as follow-up CFS (Spearman r = 0.22, p < 0.01) and KPS (Spearman r = − 0.27, p < 0.001).

In 232 patients (84%), tumors were found to be IDH wildtype and 44 (16%) revealed IDH mutation. Patients with IDH mutation showed significantly better KPS scores preoperatively and at the follow-up visits (p < 0.001 for each). There was a significant association between mutated IDH and better preoperative (p = 0.025) and follow-up CFS (p = 0.05) as well. In 146 patients (54%), MGMT promotor was methylated. There was no significant association with preoperative or follow-up scores. The detailed results are shown in Table 1.

**Table 1:** The Rockwood Clinical Frailty Scale (CFS) and Karnofsky Performance Score (KPS) before and 3–6 months after resection are shown as median with interquartile ranges according to IDH and MGMT promotor status

	CFS: pre-operatively	*p*	CFS: follow-up	*p*	KPS: pre-operatively	*p*	KPS: follow-up	*p*
IDH								
Mutated	2 (2–3)	0.025	2 (2–3)	0.05	90 (90–100)	< 0.001	95 (90–100)	< 0.001
Wildtype	3 (2–4)		3 (2–4)		80 (80–90)		80 (50–100)	
MGMT promotor								
Methylated	3 (2–4)	n.s	3 (2–3)	n.s	90 (80–90)	n.s	90 (70–100)	n.s
Unmethylated	3 (2–4)		3 (2–4)		90 (80–90)		80 (50–100)	

**Fig. 3:**
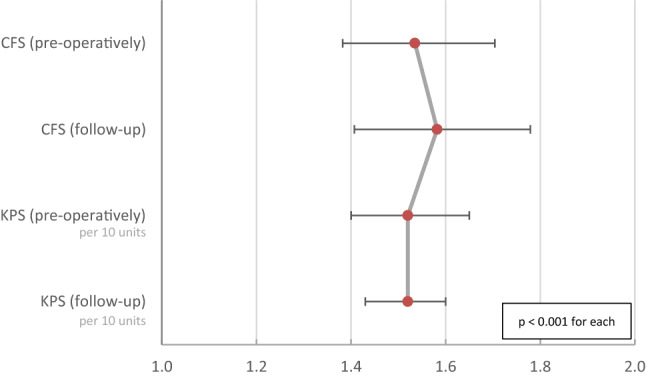
OS reduction after one point gain in the Rockwood Clinical Frailty Scale (CFS) or reduction of 10 units in Karnofsky Performance Score (KPS) before the resection (preoperatively) and 3–6 months afterwards (follow-up) is shown as hazard ratios with CI95% according to the individual Cox regression models

**Fig. 4:**
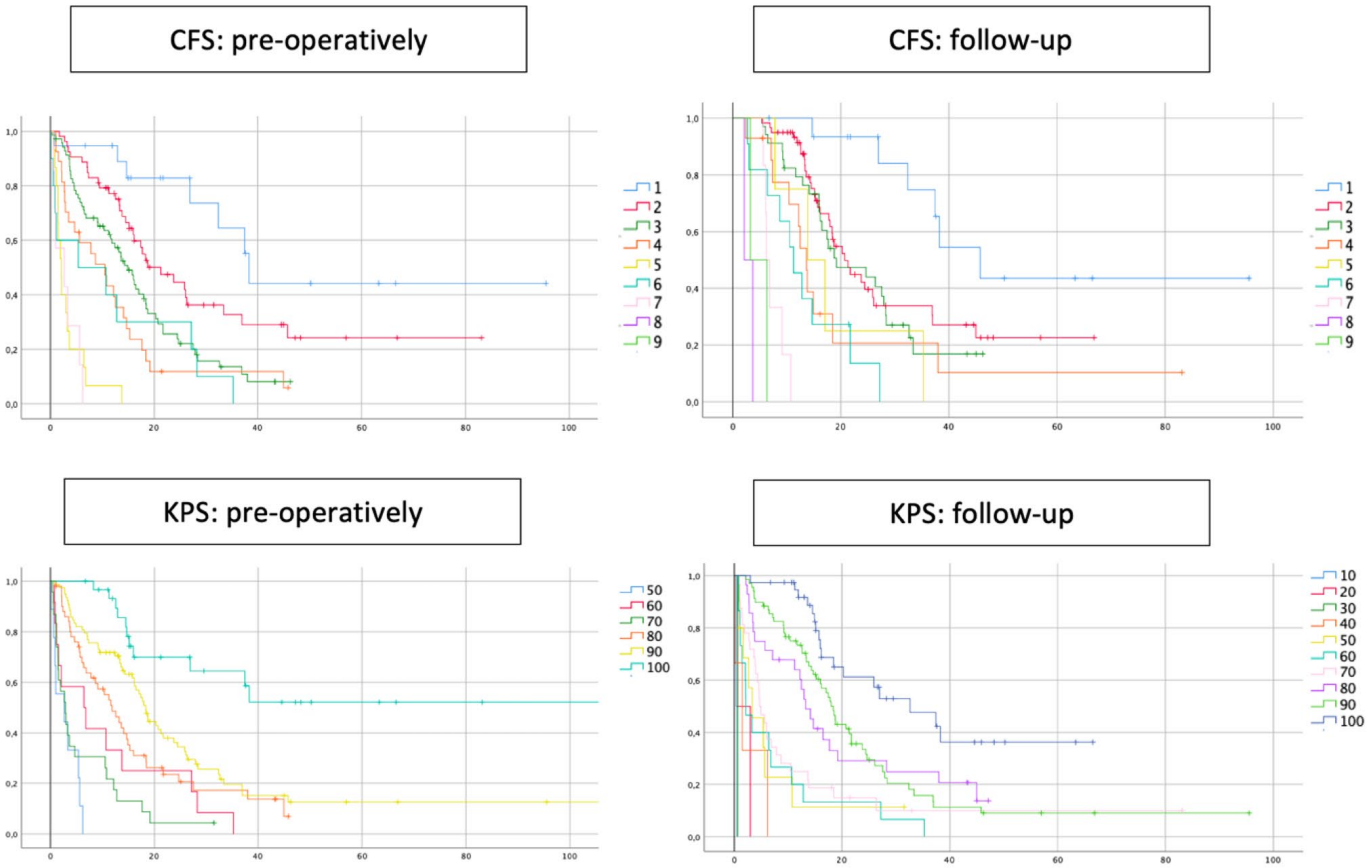
Kaplan–Meier graphs for OS considering Rockwood Clinical Frailty Scale (CFS) and Karnofsky Performance Score (KPS) points before the resection (pre-operatively) and 3–6 months after (follow-up)

**Fig. 5:**
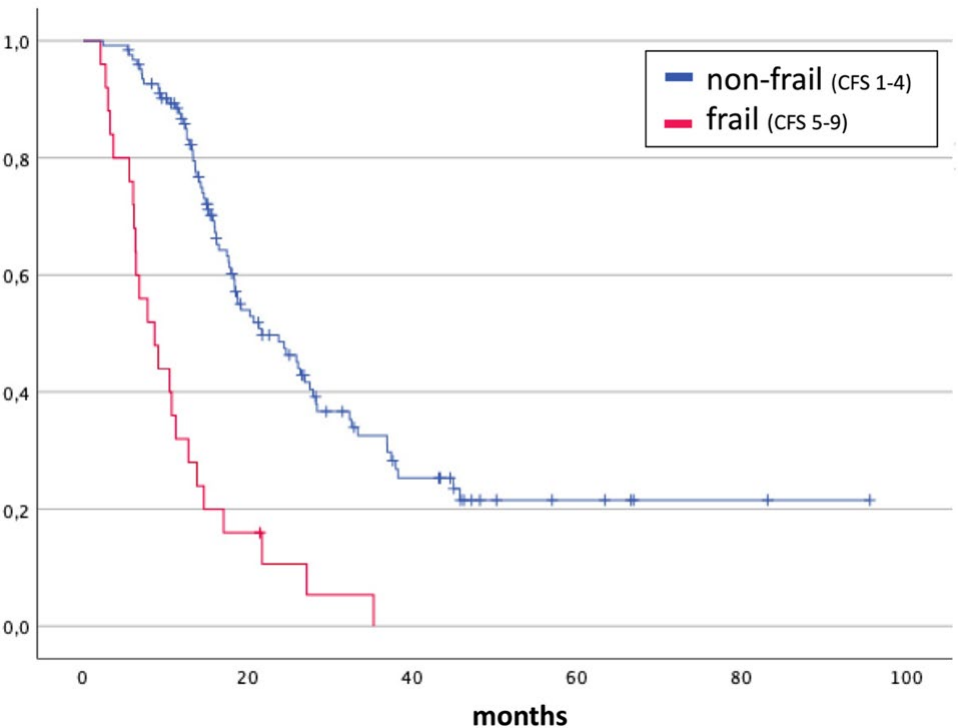
Kaplan–Meier graphs for OS considering Rockwood Clinical Frailty Scale (CFS) 3–6 months after surgery. Patients were grouped according to CFS in non-frail (CFS 1–4) and frail (CFS 5–9). LogRank test p < 0.001

According to the Cox regression (Fig. 3), the overall survival (OS) was reduced by 54% (HR 1.54, CI 95% 1.38–1.70; p < 0.001) for each additional point in preoperative CFS. At the 3 to 6 months follow-up, per point increase in CFS came along with a reduction of OS by 58% (HR 1.58, CI 95% 1.41–1.78, p < 0.001). For KPS, a reduction of OS by 52% per 10 units’ deficit in preoperative KPS (HR 1.52, CI 95% 1.40–1.65; p < 0.001) as well as during follow-up (HR 1.52, CI 95% 1.43–1.60; p < 0.001) was revealed. The detailed OS shown as Kaplan–Meier curves per CSF point or KPS step can be found in Fig. 4. The distinction between ‘frail’ (CFS 5–9) and ‘non-frail’ (CFS 1–4) patients depicts the OS as significantly different (Fig. 5). Preoperative frail patients showed a median OS of 2.0 months (± 0.7, p < 0.001) compared to 12.0 months (± 1.0, p < 0.001) in non-frail patients. At the time of follow-up, the remaining patients classified as frail showed a median OS of 7.0 months (± 1.4, p < 0.001) as opposed to the non-frail patients with a median OS of 15.0 months (± 1.2, p < 0.001).

## Discussion

This study revealed a high correlation between CFS and KPS scales in patients with high-grade glioma pre-operatively as well as at the 3–6 months follow-up. Both poorer CFS and KPS had a significant negative impact on the overall survival: the hazard to die increased by over 50% per step of each scale. IDH mutation indicates better performance before surgery and 3–6 months postoperatively. Individual performance only mildly correlated with age; moreover, OS was impacted by the performance independently from age.

Frailty describes individual vulnerability, independent of current diagnosis. It includes an extensive range of limitations that play a role in a patient`s physical and mental state. Hence, it determines the personal quality of life and overall wellbeing. It was described that prediction of functional outcome after intracranial tumor surgery was very complex, even if machine learning algorithms were used [21]. On the other hand, frailty showed an association with complications and mortality, transfer to a higher level of care facility, length of hospital stay, re-operation and re-admission across other fields of neurological surgery [22]. In other medical specialties, frailty has served as a reliable predictor of patient morbidity and mortality. In the past ten years, more than 500 articles about the influence of CFS on patient survival have been published. Since it is based on clinical judgement and consists of few and easily differentiable levels (Fig. 1), it is comfortably applicable throughout all specialties.

In our study, KPS and CFS highly correlated (r > 0.85) with each other preoperatively and at the follow-up 3–6 months after the surgery. There is a strong but not absolute correlation between the two score systems; therefore, reliability of CFS can be compared to that of KPS, but still we noted some slight differences. Moreover, the hazard to decease raised equally by about 50% within 10 months per one step worsening of KPS and CFS. Thus, our study confirmed, that both scales are comparable and both could be applied to neuro-oncological patients. Note how the curves in Kaplan–Meier processing can be differentiated with less difficulty in CFS, especially in lower CFS grades (Fig. 4). CFS and KPS can be contributed equally, since they have strong internal correlation and similar survival HR. Previous and important studies have been using KPS for many years. We think, the clinical evaluation should not focus only on the actual impact of cancer symptoms and systemic cancer therapy, such as represented by KPS. Besides, KPS rating has been shown to vary amongst the examining physicians, depending on clinician age, specialty and familiarity with clinical trials [23]. To determine the interrater variability, CFS was retrospectively assessed by four physicians and weighted kappa test was pursued, which showed the highest level of agreement (kappa 0.81, p < 0.001). A multi-center study which investigated interrater-variability concerning CFS in ICU patients showed a high level agreement even amongst raters from different backgrounds (doctors, nurses, physiotherapists) with perfect agreement in 53% and an overall good agreement (kappa 0.74) [24]. This once again underlines the simplicity of applying CFS.

In CFS, identifies vulnerability and dependency resulting from patients’ physical and mental wellbeing. It emphasizes on non-cancer related vulnerability prior and besides the glioma diagnosis, which we think should be integrated in the clinical evaluation. Thus, implicating CFS can help to gain more complex approach to estimate functional outcome and survival. CFS helps to gain a more holistic image of a patient’s condition and can contribute to more individualised and tailored therapy plan.

Patients who present with worse performance do not only face poorer functionality and independence, but they live shorter as well. Follow-up hazard ratio values for survival were very similar to those preoperatively; therefore, estimation of overall survival can be similarly accomplished before surgery and postoperatively. Thus, patients with a poor preoperative CFS should be informed about estimated postoperative functional outcome and survival. For example, a patient with a preoperative CFS of 4, meaning they feel slowed up during the day and activities are limited by symptoms, had a 54% higher likelihood to die during the study follow-up period than a patient with CFS 3, which describes a patient whose medical problems are well-controlled while not being regularly active.

Significant dependency between IDH mutation status and better functional status of the patient before and after the surgery was confirmed. It is well known, that mutation of IDH is associated with a better survival [25, 26]. Hence, this genetic characteristic does not only predict the life expectancy of the patient but also the functionality. Thus, IDH mutated and wildtype gliomas appear as two different kinds of tumors not only from a neuropathological point of view due to different pathways at development, but also in the clinical practice predicting both survival and performance–providing a perfect example of basic knowledge translation and collaboration between preclinical and applied medical branches.

Since MGMT is mostly a predictive marker for chemosensitivity, we found no correlation of MGMT promotor methylation status and CFS or KPS. Concordantly, in an integrated Cox regression model, MGMT does in fact have a positive influence on overall survival independently from performance. That translated to longer OS in patients with MGMT promotor methylation, however, there was no link to a higher functional level of these patients.

Elderly patients often harbor co-morbidities that make them vulnerable. All of these aspects can be included in the CFS rating, not only physical non-tumor-associated impairments but also cognitive deficits, other diseases or polypharmacy should be taken into account during patient evaluation [27]. Geriatric glioblastoma patients with increased frailty have shown to be at a higher probability for poorer survival with increasing patient age [28]. Our study confirmed a significant impact on overall survival with increasing patient age independently from clinical performance. So, age does influence OS, but performance (CFS and KPS) does so as well. Our study only showed a mild correlation of age with preoperative and follow-up clinical scores. In other words, age cannot replace functional performance and both these factors should be evaluated separately. This finding discourages to use age cut-offs as decisional criteria for treatment.

Thus, to predict overall survival, an integrative analysis is indispensable. While clinical scores help depict a patient’s quality of life, histological and molecular characteristics are renowned aspects for therapy decisions. Only when regarding these aspects combined, we have the chance to ensure the best treatment for individual patients.

Limitations of the study are based on the retrospective character including the possibility for interrater variability during assessment of KPS.

## Conclusions

In this first evaluation of CFS for neuro-oncological patients, the scale has shown to be a useful and reliable tool to estimate survival in patients suffering from high-grade glioma. With inclusion of cancer-unrelated vulnerability it should be used more frequently. With similar pre- and postoperative value hazard ratios, performance-dependent survival deviations could be projected reliably ahead of surgical resection.

Patients with IDH-wildtype tumors showed not only shorter OS, but also poorer functional performance. OS is influenced by performance and patient age independently; thus, these factors must be evaluated separately as a part of an integrated preoperative assessment.

- The authors declare that no funds, grants, or other support were received during the preparation of this manuscript.
- The authors have no relevant financial or non-financial interests to disclose.
- Christian F. Freyschlag and Claudius Thomé contributed to the study conception and design. Material preparation and data collection were performed by Julia Klingenschmid, Johannes Kerschbaumer and Daniel Pinggera. Statistical analysis was performed by Aleksandrs Krigers. The first draft of the manuscript was written by Julia Klingenschmid and all authors commented on previous versions of the manuscript. All authors read and approved the final manuscript.
- The datasets generated during and analysed during the current study are available from the corresponding author on reasonable request.
